# Effect of Raw and Extruded Propionic Acid-Treated Field Beans on Energy and Crude Protein Digestibility (*In-Vitro* and *In-Vivo*), Growth and Carcass Quality in Grow-Finisher Pigs

**DOI:** 10.3390/ani11113080

**Published:** 2021-10-28

**Authors:** Alberto Torres-Pitarch, Anna M. Perez-Vendrell, Edgar G. Manzanilla, Gillian E. Gardiner, Tomas Ryan, John V. O Doherty, David Torrallardona, Peadar G. Lawlor

**Affiliations:** 1 Teagasc, Pig Development Department, Animal and Grassland Research and Innovation Centre, Moorepark, Fermoy, P61 R966 Co. Cork, Ireland; a.torres.pitarch@gmail.com (A.T.-P.); egmanzanilla@gmail.com (E.G.M.); tomas.ryan@teagasc.ie (T.R.); 2 School of Agriculture and Food Science, University College Dublin, Belfield, D04 C7X2 Co. Dublin, Ireland; john.vodoherty@ucd.ie; 3 IRTA, Animal Nutrition, Mas Bové, 43120 Constantí, Spain; Anna.perez@irta.cat (A.M.P.-V.); David.torrallardona@irta.cat (D.T.); 4 Department of Science, Waterford Institute of Technology, Co. Waterford, X91 Y074 Waterford, Ireland; ggardiner@wit.ie

**Keywords:** field beans, pigs, extrusion, digestibility

## Abstract

**Simple Summary:**

European pig meat production is highly dependent on imported soybean meal (SBM). The area of field beans produced in the EU has increased greatly in recent years. There is renewed interest in field beans as an energy and protein source. Extrusion of raw ingredients can increase their nutritional value. It was hypothesized that propionic acid-treated field beans can be used to replace SBM in finisher diets and that extrusion of field beans will improve their nutritional value. Three experiments were conducted to determine the effect of extrusion of field beans on energy and crude protein digestibility (*in-vitro* and *in-vivo*), growth and carcass quality of grow-finisher pigs. Field beans are a good energy and protein source and can be fed at up to 37% inclusion in pig diets. Extrusion of field beans increased the digestible energy (DE) value, decreased the digestible crude protein (dCP) value of field beans, and had no effect on pig growth.

**Abstract:**

The *in-vitro* ileal digestibility of dry matter (DM), organic matter (OM), and crude protein (CP) of field beans treated with propionic acid (trFB) and extruded trFB (exFB) was determined in experiment 1. The DE and dCP values of trFB and exFB were determined using the difference method in experiment 2. The effect of replacing SBM with trFB and exFB in grow-finisher diets on growth, carcass quality, apparent ileal digestibility (AiD), and total tract digestibility (ATTD) of DM, OM, gross energy (GE), and CP were investigated in experiment 3. In exp. 1, *in-vitro* digestibility of exFB compared to trFB was unchanged for DM (*p* = 0.12), increased for OM (*p* < 0.05), and increased for CP (*p* < 0.05). In exp. 2, the DE value of trFB and exFB was 14.38 and 15.75 MJ/kg respectively; and the dCP value was 21.35% and 21.42% respectively (on DM basis). In exp. 3, ADFI was higher for pigs fed trFB and exFB compared to the control diet (CON; *p* < 0.05), while ADG, FCR and carcass quality parameters of pigs did not differ among treatments (*p* > 0.05).

## 1. Introduction

Fluctuations in feed costs and the high dependency of the EU feed sector on the importation of soybean meal (SBM) drive the exploration of alternative protein sources [1,2]. Among others, the use of oilseeds, legume seeds, insect meal, and by-products of biofuel production have been investigated as alternatives to SBM in pig diets [3,4,5,6]. Due to policy support for legume crops, the area of field beans (*Vicia Faba*) produced in the EU has increased greatly in recent years; in 2018 the production of field beans in the European Union was 60% greater than that reported in 2008 (FAO, 2021). On a dry matter basis, field beans contain 23.1% (±2.2, SD), 0.94% (±0.2, SD), and 13.8 MJ/kg (±0.4, SD) crude protein (CP), ether extract (EE), and gross energy (GE), respectively [7]. Therefore, there is renewed interest in field beans as an energy and protein source for livestock. Due to the high moisture content at harvesting, raw field beans are commonly treated with propionic acid prior to storage to prevent the proliferation of yeasts and molds. Extrusion which involves thermal and mechanical treatment has been suggested as a good tool to reduce the levels of intrinsic antinutritional factors and increase the nutritional value of legumes [8,9].

In this study it was hypothesized that field beans could be used to replace SBM in diets for grow-finisher pigs. It was also hypothesized that the nutritional value of field beans can be further improved by extrusion. Three experiments were conducted with the objective of determining: (1) The effect of extruding field beans crushed and treated with propionic acid on *in-vitro* ileal digestibility of dry matter, organic matter, and crude protein; (2) the digestible energy (DE) and digestible crude protein (dCP) value of field beans crushed and treated with propionic acid (trFB) and extruded trFB (exFB); and (3) the effect of replacing SBM with trFB and exFB on growth performance, carcass quality, apparent ileal digestibility (AiD), and total tract digestibility (ATTD) of DM, OM, GE, and CP in grow-finisher pigs.

## 2. Material and Methods

**Figure 1:**
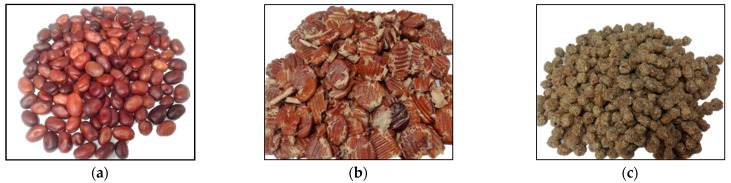
(**a**) Raw field beans (*Vicia faba*); (**b**) field beans mechanically crushed in a two-roller mill and treated with propionic acid (trFB). (**c**) Field beans mechanically crushed in a two-roller mill, treated with propionic acid and extruded (exFB) at 105 °C 5 × 5 × 6 mm die (EXTRU-tech E525, Sabetha, KS, USA).

The care and use of the animals in this study was approved by the Teagasc Animal Ethics Committee (Approval no. TAEC86/2015). The experiment was conducted in accordance with Irish legislation (SI no. 543/2012) and the EU Directive 2010/63/EU for animal experimentation. In this study, the same batch of raw field beans (*Vicia Faba*, Lynx variety) harvested in Ireland in 2016 was used. The raw field beans were mechanically crushed using a two-roller mill and treated with propionic acid prior to storage. Thereafter, a portion of this batch was extruded (105 °C, 5 × 5 × 6 mm die, EXTRU-tech E525, Sabetha, Kansas). The feeder rate was set at 450 kg/h. The temperature measured at the conditioner was 76 ± 1.5 °C, at the exit from the dye 98 ± 3.2 °C and at the dryer 62 ± 2.3 °C. The appearance of raw field beans (rwFB), trFB, and exFB are presented in Figure 1. The chemical composition of SBM, trFB, and rwFB used in this study is presented in Table 1 (*n* = 2). Laboratory analyses were performed according to the analytical methods described in Section 2.4.

### 2.1. Experiment 1

The in-vitro ileal digestibility of dry matter (DM), organic matter (OM), and crude protein (CP) of trFB and exFB was determined in experiment 1. A two-step *in-vitro* incubation procedure adapted from Boisen and Fenandez [10] and Akinsola [11] was used according to the protocol described in Torres-Pitarch et al. [12]. In short, each sample of trFB and exFB was incubated using ANKOM F-57 nylon bags in a DAISY II incubator at 39 °C (Ankom Techn., Macedon NY, USA). The first incubation step consisted of an enzymatic hydrolysis with a pepsin solution at pH 2.0 at 39 °C for 5 h. The second incubation step consisted of a hydrolysis with a multi-enzyme pancreatine (mixture of protease, amylase and lipase from porcine pancreas; Sigma-Aldrich ref. P1750, Merck KGaA, Darmstadt, Germany) at pH 6.8 and 39 °C for 17 h. Each sample was incubated in duplicate, and each incubation consisted of 22 nylon bags per incubation. Laboratory analyses were performed according to the analytical methods described in Section 2.4.

### 2.2. Experiment 2

The digestible energy (DE) and digestible CP (dCP) values of trFB and exFB were determined using the difference method [13] in experiment 2. A total of 48 male pigs [Maxgrow × (Landrace × Large White); Hermitage Genetics, Sion Road, Kilkenny, Ireland] with an initial live weight of 23.3 ± 0.66 kg (SEM) were housed in pairs. Pig pairs were allocated to 1 of 3 dietary treatments: (T1) Basal diet based on barley and soybean meal; (T2) 50% basal diet + 50% trFB; and (T3) 50% basal diet + 50% exFB. The ingredient composition and chemical analysis of the basal diet used in experiment 2 are presented in Table 2. Pairs of pigs were housed in fully slatted pens (1.81 m × 1.18 m) with steel rail partitions. Air temperature was maintained at 20 to 22 °C. The feeders were stainless steel dry feed hoppers, 30 cm in width (O’Donovan Engineering, Coachford, Co. Cork, Ireland). Ad-libitum access to feed (dry pellets) and water (one drinking bowl per pen; DRIK-O-MAT, Egebjerg International A/S, Egebjerg, Denmark) was provided. Pigs were observed closely twice daily. After 7 days of adaptation to the diets, feed and fecal samples were collected over 3 consecutive days, pooled by pen and type (feed or feces), and stored at −20 °C for later analysis (8 pens/treatment). Prior to analysis, feed and fecal samples were thawed and dried to a constant weight at 55 °C for 72 h. After drying, samples were individually ground through a 1 mm screen using a CyclotecTM mill (FOSS electric, Hilleroed, Denmark). Each sample was analyzed for DM, CP, GE and acid insoluble ash (AIA) as inert marker for the determination of DE and dCP. The DE and dCP was calculated following the equation described in Zhang and Adeola [13]. The digestibility of the test ingredient (Dti) is calculated as follows:

Dti = Dbd + ((Dtd − Dbd)/Pti)

where Dbd = digestibility basal diet, Dtb = digestibility of the test diet, and Pti = proportion of the test ingredient. Laboratory analyses were performed according to the analytical methods described in Section 2.4.

### 2.3. Experiment 3

The effect of replacing SBM with trFB and exFB in grow-finisher diets on growth performance, carcass quality, apparent ileal digestibility (AiD), and total tract digestibility (ATTD) of DM, OM, GE, and CP was investigated in experiment 3. A total of 60 pigs [Maxgrow × (Landrace × Large White); Hermitage Genetics, Sion Road, Kilkenny, Ireland] with an initial live weight of 46.2 ± 0.52 kg (SEM) were housed in same sex pen groups of 2 pigs. The pens were blocked on the basis of sex (female and entire male) and initial body weight and assigned to 1 of 3 dietary treatments in a randomized block design (n = 10 pens/treatment; 5 pens with entire male and 5 pens with females per treatment). The dietary treatments were: (T1) control diet (CON), (T2) diet with an inclusion rate of 40% of trFB (trFBD), and (T3) diet with an inclusion rate of 36.8% of exFB (exFBD). The different inclusion levels for trFB and exFB were due to the higher DM content of exFB compared to trFB (87.85% vs. 80.75%); the inclusion of trFB and exFB on a DM basis was 32.3% in T2 and T3, respectively. The ingredient composition and chemical analysis of the dietary treatments used in experiment 3 are presented in Table 2. Pairs of pigs were housed in fully slatted pens (1.81 m × 1.18 m) with solid plastic partitions at the Teagasc Pig facilities (Moorepark, Fermoy, Co. Cork, Ireland). Air temperature was maintained at 20 to 22 °C. The feeders were stainless steel dry feed hoppers, 30 cm in width (O’Donovan Engineering, Coachford, Co. Cork, Ireland). Ad-libitum access to feed (dry pellets) and water (one drinking bowl per pen; DRIK-O-MAT, Egebjerg International A/S, Egebjerg, Denmark) was provided.

The experiment lasted 63 days during which live weight and feed intake were recorded every 2 weeks and average daily gain (ADG), average daily feed intake (ADFI), and feed conversion ratio (FCR) were calculated. Fresh feces and feed samples from the feeder were collected on days 61 and 62 and stored at −20 °C for subsequent ATTD determination (6 pairs/treatment were randomly selected). At day 63 of the experimental period, pigs were transported to a commercial abattoir (Dawn Pork and Bacon, Waterford, Ireland), stunned with CO_2_, and killed by exsanguination. At slaughter, hot carcass weight was recorded, and back-fat thickness and muscle depth, measured at 6 cm from the edge of the split back at the level of the 3rd and 4th last rib, were determined using a Hennessy Grading Probe (Hennessy and Chong, Auckland, New Zealand). Lean content was estimated according to the following formula (S.I. No. 413/2001—Department of Agriculture Food and Rural Development, Republic of Ireland): Estimated lean meat content (%) = 60.3 − 0.847x + 0.147y where x = fat depth (mm); y = muscle depth (mm). At slaughter, the intestinal tracts of 36 pigs (6 pairs/treatment) were recovered. Digesta samples were collected from the terminal ileum (up to 1.5 m proximal to the ileo-caecal valve) and stored at −20 °C for later AiD determination [14]. Feed, feces, and ileal digesta samples were freeze-dried and individually ground through a 1-mm screen using the CyclotecTM mill. After milling, each sample type was pooled by pen (*n* = 6 per treatment) and analyzed for DM, ash, AIA, GE, and CP for determination of AiD and ATTD using the analytical methods described in Section 2.4 laboratory analysis.

### 2.4. Laboratory Analysis

Dry matter was determined according to the method AOAC.934.01 of the Association of Official Analytical Chemists (AOAC, 2005). The GE content was determined using an adiabatic bomb calorimeter (Parr Instruments, Moline, IL, USA). The CP content (N × 6.25) was determined using the LECO FP 528 instrument (Leco Instruments UK LTD., Cheshire, UK) according to the method AOAC.990.03. The concentration of AIA was determined according to [15]. Amino acid concentration were determined using high-performance liquid chromatography [16]. Ether extract was determined according to the method described by Usher et al. [17] by extraction with perchlorethylene in a Foss Let 15,300 (A/S N. Foss Electric, Hillerod, Denmark). The trypsin inhibitor activity and tannic acid equivalents were determined using method num. S1196 and S1166 respectively by Sciantec Ltd. (Stockbridge Technology Centre Cawood, North Yorkshire, UK).

### 2.5. Statistical Analysis

All data were analyzed using the MIXED procedure of SAS^®^ software version 9.4 (SAS Institute, Inc., Cary, NC, USA). In experiment 1, type of field beans (trFB or exFB) was included in the model as a fixed effect and the incubation batch was included in the model as a random effect. The model used in experiment 1 stands as follows:

y = μ + d + s + d × s + i + e

where y is the variable of study, d is the effect of diet, s is the effect of sex, i is the incubation batch, and e is the error term. In experiment 2, the mean value for the determination of DE and dCP was reported and no statistical comparisons were made. In experiment 3, dietary treatment, sex, and their interaction were included in the models as fixed effects for the analysis of LW, ADG, ADFI, and FCR, carcass yield, muscle depth, backfat, and lean meat percentage. Initial LW was included as a covariate in the model for growth parameters and hot carcass weight for carcass quality parameters. Pen was regarded as the experimental unit, and block was included in the models as a random effect. The model used in experiment 3 stands as follows:

y = μ + d + s + d × s + b + e

where y is the variable of study, d is the effect of diet, s is the effect of sex, b is the block, and e is the error term. A compound symmetry covariance structure was fitted to all data. Model suitability was investigated by checking normality of scaled residuals using the Shapiro–Wilk test within the UNIVARIATE procedure of SAS. The results are presented as least square means ± SEM. Differences in least square means were investigated using the *t*-test after Tukey-Kramer adjustment for multiple comparisons. Significance is reported for *p* ≤ 0.05 and tendencies toward significance are reported for *p* ≤ 0.10.

## 3. Results

### 3.1. In-Vitro Digestiblility (Experiment 1)

The *in-vitro* digestibility results of trFB and exFB are presented in Table 3. The in-vitro digestibility of exFB compared to trFB was not significantly changed for DM, and significantly increased for OM and CP.

### 3.2. Digestible Energy and Crude Protein Determination (Experiment 2)

The DE and dCP values (on DM basis) obtained for trFB were 14.38 MJ/kg and 21.4% (ATTD coefficient of CP = 78.0%), respectively. The DE and dCP values (on DM basis) obtained for exFB were 15.75 MJ/kg and 21.4% (ATTD coefficient of CP = 79.1%), respectively.

### 3.3. Growth Performance and Carcass Quality (Experiment 3)

The effect of replacing dietary SBM with trFB and exFB on AiD and ATTD of grow-finisher pigs is presented in Table 4. Dry matter and OM AiD were higher in pigs fed exFBD than pigs fed the CON diet, but similar to pigs fed the trFBD (Table 4). Dry matter, OM and CP ATTD did not differ among dietary treatments (Table 4).

The effect of replacing dietary SBM with trFB and exFB on growth performance and carcass quality traits of grow-finisher pigs is presented in Table 5. Pigs fed trFBD and exFBD had higher ADFI than pigs fed the CON diet (*p* < 0.05). Average daily gain, FCR, carcass weight, kill out percentage, fat depth, and muscle depth did not differ among dietary treatments (*p* > 0.05). There was no statistical difference in the associated interactions between dietary treatment, sex, and week.

## 4. Discussion

The trFB used in this experiment had a lower DM content (80.8 vs. 88.7%), GE (18.7 vs. 19.2 MJ/kg), and CP (27.4 vs. 53.6%) than the SBM used in this experiment (Table 1). The content of crude fiber was higher in trFB than in SBM (5.9 vs. 3.7%). Compared to the average values described in the literature for field beans grown in continental Europe [7], the trFB used in this experiment had a lower CP content (27.4 vs. 31.1%) and similar GE (18.7 vs. 18.7 MJ/kg). All values discussed above are expressed as DM basis. These results are in line with the chemical values reported in trials with field beans grown in Britain [18] where the CP value of British grown field beans is also lower than that of continental European grown field beans. The authors believe that these lower nutritional values may be related to different harvest conditions between countries, the use of varieties that are selected to have low levels of antinutritional factors, and to suit the cooler and wetter growing conditions experienced in Ireland and Britain. As expected, when trFB was extruded, the DM value and GE content of exFB were increased. The antinutritional factors analyzed [trypsin inhibitor activity (TIA) and tannin acid equivalents] for SBM, trtFB, and exFB were low and similar among trtFB, exFB, and SBM. The results obtained for TIA are in line with those obtained by [19] with a commercial, white-flowered variety of field beans. Grow-finisher pigs should not receive more than 3.0 TIU/mg in the final feed [20]. To our knowledge, maximum levels of tannins in pig diets are not described in the literature, and some recent research now consider tannin extracts as active compounds to modulate gut health in pigs rather than classifying them as antinutritional factors [21]. The tannin extracts used in monogastric nutrition are usually of seed origin (chestnuts and grapeseed) and included at between 0.05 and 5% in the diet [18]. Dietary tannins have also been found to accumulate in bone tissue, suggesting that ingredients rich in tannins may have bone-strengthening properties [22]. However, more research on the impact of dietary tannins in monogastric nutrition is needed to determine the tannin sources and concentrations most likely to show benefit in pigs.

Field beans are harvested in Ireland with a high moisture content up to and exceeding 20%. Propionic acid treatment of raw harvested field beans prior to storage is a common practice in the feed industry to allow moist storage of the field beans while preventing the proliferation of yeasts and molds during storage. This practice has also been successfully used in the storage of maize [23], wheat, and barley. Propionic acid has a disagreeable rancid odor that might be associated with lower palatability [24]. Nevertheless, studies to date, have not shown a reduction in feed intake when propionic acid was supplemented to pig diets [25]. Propionic acid has recently been considered as a feed additive to favorably modulate the gut microbiota in piglets [24,26], however the dietary concentrations used in these trials are higher than in the present study. Furthermore, the benefit of organic acids like propionic acid is less well documented in grow-finishing pigs compared to weaned piglets. An alternative to propionic acid storage of field beans is to dry the seeds prior to storage, however, in practice the hardness of dried field bean seeds is such that they can be extremely damaging to feed mill equipment such as elevators and because of this moist storage is often the preferred method of storage by the feed industry for field beans.

In this experiment, extrusion of trFB increased the *in-vitro* digestibility of OM and CP. However, this trend was not observed in the *in-vivo* experiment where nutrient digestibility values remained unchanged when diets formulated with trFB and exFB were fed to grow-finisher pigs. The *in-vitro* assay was performed on the field beans themselves while the *in-vivo* assay was performed on complete diets with an inclusion level of 32.3% (on a DM basis) of field beans. Therefore, this lack of consistency between the *in-vitro* and *in-vivo* results may be attributed to a dilution effect and possibly interactions of field beans with the other dietary components. To our knowledge, *in-vitro* digestibility of trFB or exFB has not been previously reported in the literature. In this experiment, similar ileal and total tract apparent digestibility were found in pigs fed SBM-based diets and trFB diets. This result agrees with [27] who found similar CP ATTD when replacing SBM with a 30% inclusion of raw field beans in diets for grow-finisher pigs. Feeding exFB in diets in this experiment increased the AiD of DM and OM compared to that of pigs fed SBM-based diets. Comparable digestibility values for extruded field beans could not be found in the literature.

The *in-vitro* digestibility values for field beans reported in this experiment are in line with those found in the literature. Jezierny et al. [28] reported *in-vitro* digestibility values between 77 and 94% for different varieties of field beans. The DE values generated in this experiment for trFB and exFB (14.38 and 15.75 MJ/kg, respectively) are in line with values reported in the literature [7,29]. Sauvant et al. [7] reported an average value of 16.1 MJ/kg and de Blas et al. [29] reported a DE value of 15.9 MJ/kg for field beans. The increase in DE due to extrusion is most likely due to an increase in the gelatinized starch content in the field beans in response to cooking, however this was not assessed in the current study. In line with our results, increased DE values in peas due to extrusion have previously been reported [30]. The dCP generated in this experiment for trtFB and exFB (22.8% and 21.7% respectively) are within the range of values reported in the literature [7,29]. Sauvant et al. [7] and de Blas et al. [29] reported an average dCP value for field beans of 22.24% and 20.98%, respectively. The reduced dCP value of exFB compared to trtFB reported in the current study suggests that lower processing temperatures than employed here may be advisable to minimize protein degradation during the extrusion process. The values of DE and dCP reported in the current experiment are valuable to study the effect of extrusion on field beans, nevertheless, more studies determining the Net energy (NE) and standardized ileal digestibility (SID) of amino acids are needed in order to more accurately formulate diets in an efficient and sustainable manner. In this regard, Masey O’Neill et al. [18] have reported the standardized ileal digestibility values of crude protein and amino acids of field beans for broilers but similar data are lacking for pigs.

In this study SBM was practically replaced by trFB or exFB in grow-finisher diets without impairing pig growth rate, feed efficiency, and carcass characteristics. Our results are in line with those of White et al. [28] where a 30% inclusion of low-tannin field beans successfully replaced SBM in grow-finisher pig diets without impairing growth and/or feed efficiency. However, the field bean used in that experiment were not treated with propionic acid prior to storage. In contrast to our results, Smith et al. [19] found reduced growth rates when field beans were included at 30% in finisher pig diets; tannin levels in that study were not reported. In previous studies [31,32] impaired growth and feed efficiency were reported when raw field beans were included at 12.5% and 7.5%, respectively, in grower pig diets. Trypsin inhibitor and tannin levels of the field beans varieties used in these studies [31,32], were higher than those found for the field beans used in the current study. Furthermore, in the current study field beans were treated with propionic acid previous to storage which may have had a positive effect on the nutritional value of field beans. To our knowledge, studies where propionic acid-treated field beans were used have not previously been reported in the literature. In our view, part of the success of the replacement of SBM by treated field beans may rely on the fact that the field beans in our current study were treated with propionic acid prior to storage possibly impacting positively on the growth performance results. Further research evaluating different storage conditions will be of interest for the feed industry.

In the current study, the inclusion of field beans in grow-finisher diets increased feed intake compared to SMB-based diets. The current study demonstrates that similar nutrient digestibility, growth, feed efficiency, and carcass quality traits can be achieved using locally grown field beans as a protein source for pig diets. Replacing soybean meal with field beans in grow-finisher diets will most likely have positive environmental benefits resulting from carbon saving. In order to successfully implement wider scale use of locally grown field beans in pig diets, some challenges will need to be addressed: Further research on NE and SID amino acid values are required; processing conditions need to be optimized; and storage capacity for moist propionic acid-treated beans needs to be increased; and most importantly a sustainable and consistent local supply of beans must be ensured. The volatility in feed ingredient prices, especially regarding protein sources, means that there is much more interest in ingredients, such as field beans, for inclusion in pig diets than heretofore. As grow-finisher diets are formulated on a least-cost basis the relative price/value of ingredients such as field beans compared with SBM will determine how widely they are substituted in pig diets.

## 5. Conclusions

Growth rate and feed efficiency was similar for propionic acid-treated field bean-based diets to soybean meal-based diets. Similarly, propionic acid-treated field beans either raw or extruded did not affect carcass weight at slaughter or carcass quality. Therefore, it can be concluded that propionic acid-treated field beans are a good energy and protein source for pigs that can be used to replace soybean meal in grow-finisher pig diets. Extrusion of the propionic acid-treated field beans did not improve their nutritional value for pigs and growth rate and feed efficiency was similar to that of pigs fed raw propionic acid-treated field bean-based diet.

## Figures and Tables

**Table 1:** Chemical composition of soybean meal (SBM), raw field beans treated with propionic acid (trFB) and field beans treated with propionic acid and extruded (exFB) ^1^ (*n* = 2).

	SBM	trFB	exFB
Dry Matter, %	88.70	80.75	87.85
Gross Energy, MJ/kg	19.22	18.68	18.45
Crude Protein, %	53.55	27.37	27.09
Lysine, %	2.99	1.78	1.57
Threonine, %	1.92	1.05	0.99
Methionine, %	0.72	0.22	0.22
Cystine, %	0.70	0.30	0.28
Tryptophan, %	0.77	0.27	0.25
Valine, %	2.36	1.34	1.17
Ether extract, %	2.47	1.56	1.58
Crude Fiber, %	3.72	5.94	6.15
Acid Detergent Fiber, %	5.76	9.28	9.76
Neutral Detergent Fiber,%	8.12	10.15	10.70
Ash, %	7.22	4.33	4.44
Tannic acid equivalents, mg/g	0.95	0.79	0.79
Trypsin Inhibitor Activity mg/g	1.47	1.86	1.62

^1^ Units expressed on dry matter basis.

**Table 2:** Ingredient and nutrient composition of dietary treatments of experiments 2 and 3 (in %, on as fed basis; unless otherwise specified).

	Experiment 2	Experiment 3 ^1^		
	Basal Diet	T1 (CON)	T2 (trFBD)	T3 (exFBD)
Ingredient composition				
Barley	76.29	83.02	54.40	59.60
trFB ^2^	0.00	0.00	40.00	0.00
exFB ^3^	0.00	0.00	0.00	36.80
Soybean meal	17.64	14.36	1.25	0.80
Soya oil	3.35	0.00	1.53	0.03
Limestone flour	1.10	1.12	1.20	1.15
Mono-dicalcium phosphate	0.50	0.50	0.50	0.50
L-Lysine (HCl, 78.8%)	0.38	0.35	0.26	0.27
L-Threonine (98%)	0.17	0.14	0.17	0.17
DL-Metionine	0.13	0.08	0.21	0.20
L-Tryptophan	0.01	0.00	0.05	0.05
Salt	0.30	0.30	0.30	0.30
Premix ^4^	0.10	0.10	0.10	0.10
Celite	0.03	0.03	0.03	0.03
Nutrient composition				
Dry matter ^5^	88.96	87.00	85.30	86.80
Crude Protein ^5^	16.8	15.7	15.8	15.7
SID Lysine ^6^	0.99	0.89	0.89	0.89
Lysine ^5^	1.03	0.98	0.96	0.97
Ether extract ^5^	6.0	3.1	4.5	3.5
Net energy ^6^, Mj/kg	9.7	8.9	8.9	8.9
Gross energy ^5^, Mj/kg	16.6	16.1	16.3	16.2
Total Ca ^6^	0.64	0.64	0.66	0.64
Digestible P ^6^	0.24	0.24	0.24	0.25
Crude fibre ^5^	3.6	3.5	5.4	5.8
Acid detergent fibre ^5^	4.5	4.8	7.0	7.5
Neutral detergent fibre ^5^	9.9	10.4	10.8	12.0
Ash ^5^	4.2	4.6	4.4	4.3

^1^ CON = control diet based on barley and soybean meal, trFBD = diet based on barley and propionic acid-treated field beans, rwFBD = diet based on barley and propionic acid-treated and extruded field beans. ^2^ Field beans treated with propionic acid. The inclusion rate of trFB corresponds to 32.3% in DM basis. ^3^ Field beans treated with propionic acid and extruded. The inclusion rate of trFB corresponds to 32.3% in DM basis. ^4^ Premix provided per kilogram of complete diet: Cu from copper sulphate, 15 mg; Fe from ferrous sulphate monohydrate, 24 mg; Mn from manganese oxide, 31 mg; Zn from zinc oxide, 80 mg; I from potassium iodate, 0.3 mg; Se from sodium selenite, 0.2 mg; retinyl acetate 0.7 mg; cholecalciferol, 12.5 µg; DL-alphatocopheryl acetate, 40 mg; vitamin K, 4 mg; vitamin B12, 15 μg; riboflavin, 2 mg; nicotinic acid, 12 mg; pantothenic acid, 10 mg; vitamin B1, 2 mg; vitamin B6, 3 mg. ^5^ Analyzed values (in %, on an air-dry basis; unless otherwise specified). ^6^ Calculated values (in %, on an air-dry basis; unless otherwise specified).

**Table 3:** Effect of extruding raw field beans on *in-vitro* digestibility of dry matter (DM), organic matter (OM), and crude protein (CP), (on a % DM basis) (*n* = 4).

	Dry Mater	Organic Mater	Crude Protein
trFB ^1^	73.4	73.0	90.7
exFB ^2^	74.4	74.5	91.0
Pooled SEM ^3^	0.23	0.03	0.02
*p*-value	0.12	0.02	0.04

^1^ Field beans treated with propionic acid. ^2^ Field beans treated with propionic acid and extruded. ^3^ Standard error of the mean.

**Table 4:** Effect of partially replacing dietary soybean meal with field beans treated with propionic acid (trFBD) and extruded (exFBD) on apparent ileal digestibility (AiD) and apparent total tract digestibility (ATTD) of grow-finisher pigs (*n* = 6 pairs of pigs/treatment) ^1^.

	Treatment ^2,3^				*p*-Value		
	CON	trFBD	exFBD	SEM ^4^	Diet	Sex	Diet × Sex
AiD of DM	65.4 ^a^	69.9 ^a,b^	72.3 ^b^	1.61	0.02	0.31	0.62
AiD of OM	68.5 ^a^	73.2 ^a,b^	75.2 ^b^	1.46	0.01	0.33	0.61
AiD of CP	63.7	63.3	63.3	3.20	0.39	0.66	0.83
AiD of Energy	66.1	71.0	71.2	2.18	0.33	0.44	0.77
ATTD of DM	88.3	85.9	88.7	0.29	0.13	0.26	0.88
ATTD of OM	89.9	87.9	90.1	0.27	0.15	0.31	0.46
ATTD of CP	85.2	84.0	86.2	0.57	0.33	0.34	0.72

^1^ CON = control diet based on barley and soybean meal; trFBD = diet based on raw field beans treated with propionic acid; exFBD = diet based on extruded field beans treated with propionic acid. ^2^ Values are least square means. ^3,a,b^ Values within a row that do not share a common superscript are statistically different (*p* < 0.05). ^4^ Standard error of the mean.

**Table 5:** Effect of partially replacing dietary soybean meal with field beans treated with propionic acid (trFB) and extruded trFB (exFB) on growth performance and carcass quality of grow finisher pigs (*n* = 10 pairs of pigs/treatment) ^1^.

	Treatment ^2,3^				*p*-Value		
	CON	trFBD	exFBD	SEM ^4^	Diet	Sex	Diet × Sex
Initial live weight, kg	46.2	46.2	46.2	0.52	0.98	0.72	0.77
Final live weight, kg	109.1	112.8	111.0	0.99	0.11	0.06	0.77
ADFI, g/day	2291 ^a^	2453 ^b^	2403 ^b^	43.8	0.02	0.03	0.16
ADG, g/day	998	1058	1027	22.4	0.13	<0.01	0.85
FCR, g/g	2.30	2.32	2.35	0.027	0.42	<0.001	0.15
Hot carcass weight, kg	84.4	86.9	85.7	1.13	0.12	0.21	0.76
Carcass yield, %	77.2	77.3	77.1	0.37	0.48	0.12	0.62
Fat depth, mm	11.2	12.8	12.2	0.56	0.14	0.15	0.11
Muscle depth, mm	60.7	59.7	57.8	2.62	0.47	0.35	0.68
Lean meat, %	59.9	58.4	58.3	0.78	0.16	0.23	0.14

^1^ CON = Control diet based on barley and soybean meal; trFBD = Diet based on raw field beans treated with propionic acid; exFBD = diet based on extruded field beans treated with propionic acid. ^2^ Values are least square means. ^3,a,b^ values within a row that do not share a common superscript are statistically different (*p* < 0.05). ^4^ Standard error of the mean.

## Data Availability

The data presented in this study are available on request from the corresponding author.
